# Kerboull-type plate in a direct anterior approach for severe bone defects at primary total hip arthroplasty: technical note

**DOI:** 10.1051/sicotj/2017006

**Published:** 2017-03-10

**Authors:** Mikio Matsumoto, Tomonori Baba, Hironori Ochi, Yu Ozaki, Taiji Watari, Yasuhiro Homma, Kazuo Kaneko

**Affiliations:** 1 Department of Orthopedic Surgery, Juntendo University School of Medicine 2-1-1 Hongo, Bunkyo-ku Tokyo Japan

**Keywords:** Kerboull-type plate, Direct anterior approach, Total hip arthroplasty, Technical note, Minimally invasive surgery

## Abstract

*Introduction*: For cases with extensive acetabular bone defects, we perform surgery combining the Kerboull-type (KT) plate and bone graft through direct anterior approach (DAA) in primary total hip arthroplasty (THA) requiring acetabular reconstruction as minimally invasive surgery. This paper provides the details of the surgical procedure.

*Methods*: The basic structure of the Kerboull-type plate is a cruciform plate. Since the hook of the Kerboull-type plate has to be applied to the tear drop, a space for it was exposed. The tear drop is located in the anterior lower region in surgery through DAA in supine position. It was also confirmed by fluoroscopy as needed. The bone grafting was performed using an auto- or allogeneic femoral head for bone defects in the weight-bearing region of the hip joint.

*Results*: Of 563 patients who underwent primary THA between 2012 and 2014, THA using the KT plate through DAA was performed in 21 patients (3.7%). The mean duration of postoperative follow-up was 31.8 months. The mean operative time was 188.4 min, and the mean blood loss was 770 g. The patients became able to walk independently after 2.4 days on average (1–4 days). On clinical evaluation, the modified Harris Hip Score was 45.6 ± 12.4 before surgery, and it was significantly improved to 85.3 ± 8.97 on the final follow-up.

*Discussion*: DAA is a true intermuscular approach capable of conserving soft tissue. Since it is applied in a supine position, fluoroscopy can be readily used, and it was very useful to accurately place the plate.

## Introduction

Total Hip Arthroplasty (THA) is recognized as superior in reducing pain and recovering function in patients with hip joint diseases, such as osteoarthritis of the hip, rheumatoid arthritis, and osteonecrosis of the femoral head. THA as minimally invasive surgery has recently been performed in many cases and stabilized the postoperative outcome, and shortened the duration of hospital stay [1–3]. Since the direct anterior approach (DAA) is a true intermuscular approach, it is capable of conserving soft tissue, and postoperative recovery is rapid with a low incidence of dislocation [4–6]. Thus, THA employing DAA is an ideal surgery for elderly patients in whom muscle is originally weak and the risk of dislocation is high. However, these patients include those with high dislocation due to acetabular dysplasia and those with extensive joint destruction due to rheumatoid arthritis and rapidly destructive coxarthropathy (RDC), with extensive acetabular bone defects. In these cases, strong primary fixation cannot be obtained using the conventional acetabular component alone, and acetabular reconstruction using bone graft and a reinforcement device may be effective [7]. We perform surgery combining the Kerboull-type plate and bone graft through DAA in primary THA requiring acetabular reconstruction as minimally invasive surgery to conserve soft tissue. There are several pitfalls in this surgical procedure compared with DAA using the conventional acetabular component. We report the details of this surgical procedure of acetabular reconstruction employing a combination of the Kerboull-type plate through DAA and bone graft.

## Materials and methods

### Materials

**Figure 1.**
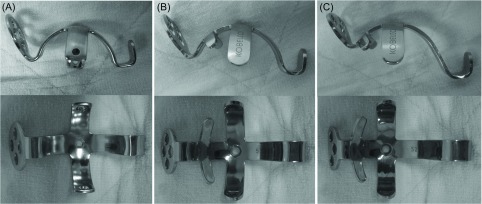
(A) Kerboull plate, (B) KT plate for placement in the original position, (C) KT plate for high placement (according to [7]).

The basic structure of the Kerboull-type plate (KT plate, Kyocera Medical Corporation, Osaka, Japan) is a cruciform plate, the same as the Kerboull plate [8], and it is a device for acetabular reconstruction (Figure 1). Centering on the dome-shaped cruciform plate to be placed in the acetabular region, it has a palette on the proximal side capable of applying acetabular screw fixation and a hook on the distal side to be set to the tear drop. It is different from the Kerboull plate in that it is made of pure titanium, there is size variation, and the shape can be changed such as the hook length and inclination angle of the palette. There are three sizes of the inner diameter of the dome: 44, 48, and 52 mm, and their outer diameters are 49, 53, and 57 mm, respectively. These are made to fit Asians with a small physique. The hook lengths are 0 mm (for positioning to the original acetabular site), and 10 mm and 15 mm (only for the device with an inner diameter of 48 mm), and it is applicable for slightly high placement. There are three types of plates with an angle of inclination of 5 and 10° (only for the device with an inner diameter of 48 mm) and 15°. The plate shape is oval, and the number of screw holes is four for the original acetabular positioning type and three for high placement. The screws are titanium alloy-made cortex screws with a diameter of 4.5 mm and a length ranging from 26 to 64 mm.

The cup was cemented directly onto the KT plate, and either a cemented or cementless stem can be placed. In addition, several specific retractors are necessary for DAA.

### Approach and surgical technique

#### Surgical procedure until reaching the hip joint

**Figure 2.**
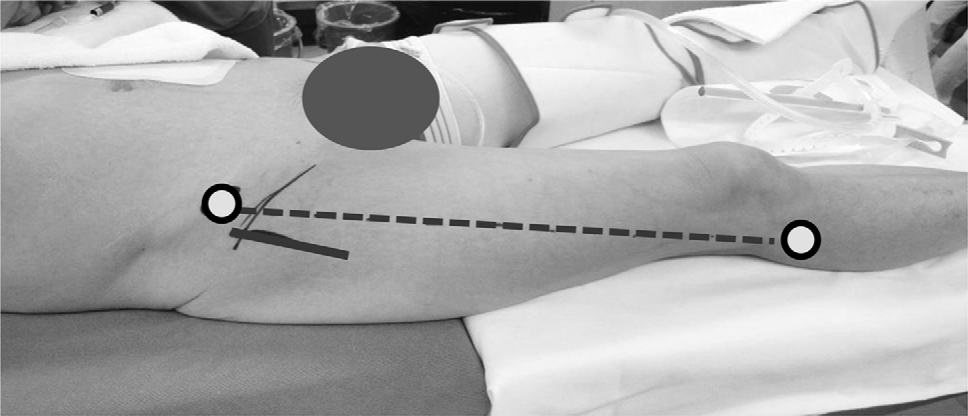
The skin incision of DAA was made from a site two-finger-breath distal and two-finger-breath lateral to the anterior superior iliac spine. It was parallel to a line drawn from the anterior superior iliac spine to the fibular head, and the incision length was about 10 cm.

A skin incision was made from a site two-finger-breath distal and two-finger-breath lateral to the anterior superior iliac spine. It was parallel to a line drawn from the anterior superior iliac spine to the fibular head, and the incision length was about 10 cm, being slightly longer than that in the conventional primary THA (Figure 2). The skin and subcutaneous tissue were incised and the fascia of the tensor fascia lata muscle was reached. The fascia was incised at a site slightly lateral to the region between the sartorius and tensor fascia lata muscles in order to prevent damaging the lateral femoral cutaneous nerve. The incised medial fascia was held with forceps, and the region between the sartorius and tensor fascia lata muscles was bluntly dissected using an elevator or finger. When a retractor was applied to the intermuscular region, the presence of the rectus femoris muscle and lateral circumflex femoral artery directing to the hip joint on the medial side can be confirmed. This artery was ligated and cut beforehand. Hoffman hooks were put on the regions between the lateral side of the femoral neck and gluteus medius and minimus muscles and between the medial side of the neck and iliopsoas muscle, respectively. By re-setting the retractor to the rectus femoris muscle and excluding the muscle medially, the anterior surface of the joint capsule was exposed. The joint capsule was incised in a V-shape and turned over. Thread was applied to the joint capsule, and Hoffman hooks were placed on the medial and lateral sides of the neck. The trochanteric tubercle, which is the joint capsule attachment site, is present in the region between the neck and greater trochanter, and the lesser trochanter is present on a line extended from this. The trochanteric tubercle was palpated with a finger, and a mark was applied toward the approximately 15° femoral head side, and osteotomy was applied. When removal of the femoral head is difficult, it can be readily removed after resection of the articular lip within a visible range and complete division of the joint capsule around the neck. The acetabular visual field was secured by re-setting the retractor to the joint capsule anterior to the acetabulum and the joint capsule incised in a V-shape. The residual joint lip was then resected. For cases with severe femoral shortening or anteversion of the neck, femoral preparation was performed in this step in advance in order to secure the acetabular visual field and make the operation easier and confirm total anteversion of the cup and stem. The pubofemoral ligament was partially cut to release limited external rotation of the hip joint. A single blunt hook was applied to the medial region of the osteotomized femoral neck to elevate it, and the posterior lateral joint capsule was divided. A retractor was set to the greater trochanter to elevate the proximal femur, and it was confirmed that reliable elevation of the greater trochanter over the acetabular posterior margin is possible. After completion of this step, the procedure returned to acetabular operation.

#### Surgical procedure of acetabular reconstruction using the KT plate

**Figure 3.**
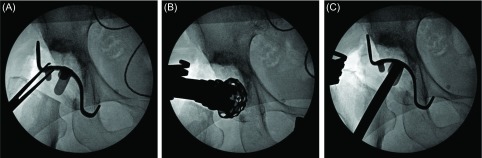
(A) The template of KT plate was placed, and the plate size and amount of the defect were confirmed. (B, C) When the template could not be sufficiently inserted, the osteophyte and acetabular floor region interfering with the template were trimmed using a small acetabular reamer, and fitting was re-confirmed.

Since the hook of the KT plate has to be applied to the tear drop, a space for it was exposed. The tear drop is present in the lower deep layer in the surgical field when surgery is performed through an approach applied in lateral recumbency, but it is located in the anterior lower region in surgery through DAA. Thus, its exposure is relatively easy. When a sufficient space for applying the hook could not be secured, the transverse ligament was partially resected. A template with a planned size was placed, and the plate size and amount of the defect were confirmed (Figure 3A). It was also confirmed by fluoroscopy as needed. When the template could not be sufficiently inserted, the osteophyte and acetabular floor region interfering with the template were trimmed using a rongeur and small acetabular reamer, and fitting was re-confirmed (Figures 3B and 3C).

**Figure 4.**
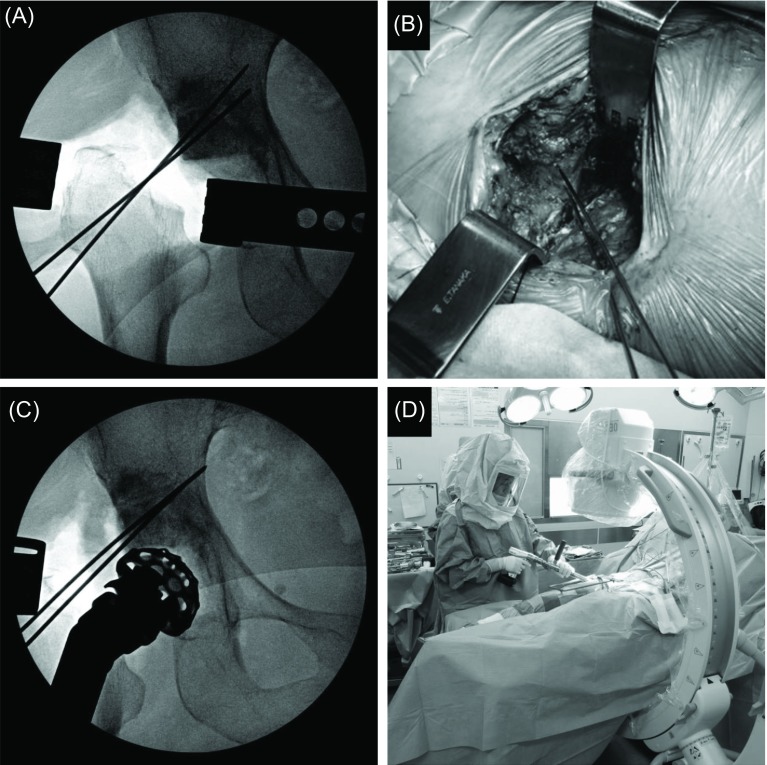
(A, B) For bone defects in the weight-bearing region of the hip joint, autologous femoral head was trimmed, and temporary fixation with it as bulk bone was applied using Kirschner wire. (C, D) Since the KT plate is dome-shaped, it is necessary to remove an excess portion of the temporarily fixed bone graft using a slightly smaller reamer. It was also confirmed by fluoroscopy as needed.

**Figure 5.**
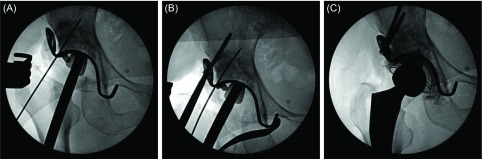
(A, B) After confirming that the KT plate was placed at an appropriate position, the retractor was set on the hook, and the plate was firmly pressed and attached to the tear drop. (C) A polyethylene liner with an optimum size was fixed with cement.

For bone defects in the weight-bearing region of the hip joint, autologous femoral head, from which cartilage was removed, was trimmed, and temporary fixation with it as bulk bone was applied using Kirschner wire (when sufficient bone strength could not be retained due to the presence of a large bone cyst in the autologous femoral head, allogeneic bone was used) (Figures 4A and 4B). When a medial acetabular defect was present, it was filled with a plate-shaped bone graft. Since the plate is dome-shaped, it is necessary to remove an excess portion of the temporarily fixed bone graft using a slightly smaller reamer (Figures 4C and 4D). When the size became optimum, the true KT plate was placed (Figure 5A). After confirming that the KT plate was placed at an appropriate position, the retractor was set on the hook, and the plate was firmly pressed and attached to the tear drop (Figure 5B). By driving the center of the KT plate, the space between the bone graft and host bone was pressed. The plate was temporarily fixed with Kirschner wire through the screw insertion region of the plate. Screws were inserted into the ilium through the plate to fix it. When one screw was fixed, the absence of instability of the plate and bone graft was confirmed. When the plate and bone graft are unstable, it is likely that: (1) the plate position is inappropriate, or (2) the position or size of the bone graft is inappropriate. The cause was investigated, and the procedure was redone. When instability was not noted, one or two screws were additionally inserted. The residual bone defect was filled with morselized bone as much as possible. A polyethylene liner with an optimum size was fixed with cement targeting a lateral opening angle of 40° and anterior opening angle of 20° (Figure 5C).

#### Insertion of femoral component

Preparation for elevation of the femur was described above. For the femoral component, either uncemented or cemented stem was selected depending on the bone quality and femoral shape. The hip joint region was extended on the operating table, setting the hip joint position to 15° extension, 90° external rotation, and 15° adduction, and preparation for stem insertion was performed. A trial broach with the optimum size was inserted, and reduction was applied. After confirming a sufficient range of motion of the hip joint and stability, an implant with an appropriate size was inserted. The joint capsule incised in a V-shape was sutured, a drain was placed, and the incised fascia and subcutaneous tissue were sutured in each layer to close the wound.

#### Postoperative Management

An antibiotic was administered at the time of the introduction of anesthesia and at 3-hour intervals thereafter (total: three times) on the day of the operation. The drain was removed one day after the operation, and edoxaban as an anticoagulant for deep veins was administered at an appropriate dose, according to the body weight and renal function, for 14 days. Range of motion training was initiated immediately after surgery. Ambulation was permitted on the day following surgery, and weight-bearing was not restricted. Training of gait using a T-cane or independent walking was performed as soon as possible. The patients were discharged when they became able to step up and down stairs. The patients visited the hospital for examination one month after discharge, and underwent plain X-ray examination and clinical evaluation at three-month intervals for one year, and at six-month intervals thereafter.

#### Clinical results

Of 563 patients who underwent primary THA between January 2012 and December 2014, THA using the KT plate through DAA was performed in 21 patients (3.7%). They were all female. Of cases of RDC, acetabular dysplasia-induced high dislocation, and RA, mainly, those with a very large acetabular bone defect and difficult to reconstruct with the conventional acetabular component alone were indicated for this procedure. All surgeries were performed by T.B. or under direct guidance by T.B. The mean age was 73.2 years old (55–83 years old), the mean height was 146.6 cm (139–155 cm), the mean body weight was 46.4 kg (41–56 kg), BMI was 21.5 (19.2–23.6 kg/cm^2^), and the mean duration of postoperative follow-up was 31.8 months (23–57 months). The disease was RDC in nine patients, severe development dysplasia of the hip (Crowe three in all) in ten, and RA in two. The mean operative time was 188.4 min (120–290 min), and the mean blood loss was 770 g (400–1,180 g). The patients became able to walk independently after 2.4 days on average (1–4 days). No serious complications, such as dislocation, infection, or fatal deep venous thrombosis, occurred in any patient after surgery using this procedure. For transfusion, autologous blood (800 g) drawn before surgery and blood collected during surgery were used, and none of the patients required allogeneic blood transfusion. On clinical evaluation, the modified Harris Hip Score [9] was 45.6 ± 12.4 before surgery, and it was significantly improved to 85.3 ± 8.97 on the final follow-up (Student’s *t*-test, *P* < 0.001).

## Discussion

According to the report of the clinical outcomes of the Kerboull plate, which is the original of the Kerboull-type plate used by us, the developers of the plate, Kerboull et al. [8], performed surgery through the lateral approach by cutting the greater trochanter in lateral recumbency. Studies using conventional approaches, such as an approach following the original method [10], posterior approach [7], and anterolateral approach [11], have occasionally been reported. However, no study has closely described the surgical procedure of acetabular reconstruction using the Kerboull-type plate through DAA which pays attention to MIS. We also employed the lateral or posterior approach by cutting the greater trochanter in lateral recumbency early after introduction of this plate [7, 12]. Based on this experience, invasiveness for soft tissue is not small in surgery employing either approach. Moreover, failure of bone union due to division of the greater trochanter is of concern for the lateral approach, and dislocation due to division of the short external rotators is of concern for the posterior approach [7, 10–13]. On the other hand, DAA is a true intermuscular approach capable of conserving soft tissue, being low-invasive. Since it is applied in a supine position, fluoroscopy can be readily used, and it was very useful to accurately place the plate [14].

For acetabular reconstruction in primary THA for cases accompanied by a large bone defect or fragile bone, one of the following procedures is selected: (a) Reconstruction with the acetabular component alone, (b) bone graft followed by placing the acetabular component, (c) acetabular reconstruction by combination of bone graft and reinforcement device, followed by placement of the acetabular component. When it is reconstructed with the acetabular component alone, problems with primary fixation ability and gait due to excessively high placement [15] and a lack of bone stock for reoperation for future revision surgery are of concern. When bone bone graft is applied followed by placing the acetabular component, excessively high placement can be corrected, and recovery of bone stock can be expected. However, the mid-to-long-term outcomes are still controversial [16–20]. Thus, we adopt the method of combining bulk bone graft and the reinforcement device following acetabular reconstruction in revision surgery. We use the Kerboull-type plate as a reinforcing device because a high long-term survival rate of patients treated with revision of the acetabulum with a large bone defect has already been demonstrated [21]. Combination of this plate and sufficient bone graft disperse the load on the acetabulum, which facilitates early weight-bearing.

## Conclusion

We reported the details of the surgical procedure of acetabular reconstruction using the Kerboull-type plate through DAA for cases with an extensive acetabular bone defect. DAA is a true intermuscular approach capable of conserving soft tissue, and it is low-invasive. By concomitantly using fluoroscopy, the Kerboull-type plate could be placed at the accurate position, and the clinical outcomes were also favorable.

## Conflict of interest

The authors declare that they have no conflict of interest.
